# Original article title: "Comparison of therapeutic efficacy of topical corticosteroid and oral zinc sulfate-topical corticosteroid combination in the treatment of vitiligo patients: a clinical trial"

**DOI:** 10.1186/1471-5945-11-7

**Published:** 2011-03-31

**Authors:** Reza Yaghoobi, Mohammad Omidian, Nooshin Bagherani

**Affiliations:** 1 Department of Dermatology, Jundishapur University of Medical Sciences, Ahvaz, Iran

## Abstract

**Background:**

Vitiligo is the most prevalent pigmentary disorder which occurs worldwide, with an incidence rate between 0.1-4 percent. It is anticipated that the discovery of biological pathways of vitiligo pathogenesis will provide novel therapeutic and prophylactic targets for future approaches to the treatment and prevention of vitiligo. The purposes of this study were evaluating the efficacy of supplemental zinc on the treatment of vitiligo.

**Methods:**

This randomized clinical trial was conducted for a period of one year. Thirty five patients among 86 participants were eligible to entrance to the study. The patients in two equal randomized groups took topical corticosteroid and combination of oral zinc sulfate-topical corticosteroid.

**Results:**

The mean of responses in the corticosteroid group and the zinc sulfate-corticosteroid combination group were 21.43% and 24.7%, respectively.

**Conclusion:**

Although, the response to corticosteroid plus zinc sulfate was more than corticosteroid, there was no statistically significant difference between them. It appeared that more robust long-term randomized controlled trials on more patients, maybe with higher doses of zinc sulfate, are needed to fully establish the efficacy of oral zinc in management of vitiligo.

**Trial Registration:**

chiCTRTRC10000930

## Background

Vitiligo has been known for thousands of years because of its visually phenotype [1,2]. It is characterized by acquired, idiopathic, progressive, circumscribed hypomelanosis of the skin and hair, with total absence of melanocytes microscopically [3].

Vitiligo is the most prevalent pigmentary disorder, occurs worldwide [4], with an incidence rate between 0.1-2% [4-8], irrespective of age, race [4,7-9], ethnic origin, or skin color [10]. Both sexes are equally afflicted [4]. In some studies, a female preponderance has been reported [2,4,11], but the discrepancy has been attributed to a presumed increase in reporting of cosmetic concerns by female patients [4]. Vitiligo commonly begins in childhood or young adulthood [4,12], with peak onset of 10 to 30 years [4,13], but it can develop at any age [4,14-17].

It is generally agreed that there is an absence of functional melanocytes in vitiligo skin and that this loss of histochemically recognizable melanocytes is the result of destruction [18]. The etiopathogenesis of vitiligo is complex, and includes genetic factors, autoimmune process, infectious factors, and psychological factors (stress and personality characteristics of patients) [19].

Zinc is one of the important trace elements related to health and disease [20]. Zinc in combination with other micronutrients such as copper, cobalt, nickel, iron, manganese, and calcium [21] plays an important role in the process of melanogenesis [3,21]. With searching the computerized bibliographic database Pub Med, we found no study of zinc efficacy in treatment of vitiligo, which motivated us to carry out this study.

## Methods

This clinical trial was conducted for a period of one year from March of 2008 till March of 2009. Eighty six vitiligo patients from 102 patients who attended the Dermatology Center of Jundishapur University of Medical Sciences participated in the study. The Jundishapour University of Medical Sciences Ethical Committee permission was obtained before performing the study. The informed consent was prepared including the definition of vitiligo, traditional therapeutic approaches and their efficacy and safety, the process of trial and the probable complication of zinc as a therapeutic new approach. According to this consent, the patients could deny the study whenever the drug complication was intolerable for them.

At first, a questionnaire was completed for each patient, which included the data of demographic status, duration of vitiligo, medical and drug history, familial status for vitiligo and pregnancy status in females. Then, for all participants, laboratory tests were recommended which comprised complete blood count and differentiation of white blood cells, fasting blood sugar, serum calcium, phosphorus and zinc levels, liver function (AST, ALT, Alk Ph and Bil), renal function (BUN, Creatinin), and thyroid function tests (T3, T4, TSH and T3RUP), urinalysis and stool examination.

In the second step, among the patients, the eligible ones who had inclusion criteria (Table. 1), were selected; The eligible patients for continuing the second step were randomized in two treatment groups. The first group took topical corticosteroid as 0.05% clobetasol propionate cream in isopropyl alcohol 65° preparation (in equal proportion) for the body and 0.1% triamcinolone acetonide cream for the face and flexures, two times daily. For the second group, topical corticosteroid (compatible with the first group) admixed with oral zinc sulfate (220-mg capsule) in dose of 2 capsules per day in teenager and adults and 10 mg/kg of capsule or syrup for children, were prescribed. For the second group, serum zinc level was repeatedly measured 1 and 3 months after commencing the treatment.

**Table 1:** The inclusion criteria for entrance to the study

Any age
Any gender
The localized and gneralized types of vitiligo with exception of segmental one (with involvement <20% of body surface)
Vitiligo duration less than 5 years
Negative history of systemic disorder
No pregnancy
Negative history of drug administration
Normal or low serum zinc
Normal other laboratory tests
Taking no zinc during 4 weeks prior to referring

All patients were assessed 1, 3 and 4 months after beginning the treatment. For comparing, we considered the largest patch as the target lesion. This target patch was selected in the way that lesions in exposed area and distal parts of limbs were not included as target lesions; so we omitted the probable bias in evaluation of response regarding to probable more rapid response in exposed areas or slower response in the hairless areas of extremities. The surface of the target lesion was measured by two physicians with a crossed sheet and a photograph was prepared for the next comparing. At the next stages, we determined the response rate regarding to the size of the target lesion.

Eventually, using the software of SPSS (Version 15), results were analyzed. P value < 0.05 was considered to be statistically significant.

## Results

A total of 86 patients with vitiligo were studied. Among these patients, 39 (45.3%) were female and 47 (54.7%) were male. Totally, 39 (45.3%) of the patients had abnormal laboratory tests, who were excluded from continuing study. The serum zinc were increased in 4 (4.7%) patients, and decreased in 9 (10.5%) patients.

According to the results with considering the inclusion criteria, out of the 86 patients, 35 were eligible for continuing the study. Then, the patients were divided in two groups, randomly; randomization in the two groups of therapy and control was performed by computerized number tables. The first group receiving topical corticosteroid included 16 (45.7%) subjects, and the second group receiving topical corticosteroid plus oral zinc sulfate was consistent of 19 (54.3%) subjects.

Considering the two treatment groups based on the sex frequency, using Pearson Chi-Square test with P-value of 0.45, showed no statistically significant difference.

The minimum, maximum and mean of age in the first group were 13.0, 57.0 and 32.2 (± 12.58), respectively, and for the second group were 11.0, 59.0 and 30.5 (± 12.11), respectively. Comparing the two treatment groups, in the view of age, with T-test and P-value of > 0.05 showed no statistically significant difference.

In the aspect of vitiligo involvement, using T-test and P-value of 0.8, no significant difference was seen between the two groups. The mean of involvement was 11.0% (± 6.6%) of body surface in the first group, whereas was 10.6% (± 8.1%) of the body surface in the second group.

In the first group, one patient (6.3%), and in the second group also one patient (5.3%) showed decreased serum zinc level. To compare the two groups in the view of serum zinc level, there was no statistical significance according to Fisher's exact test and P-value of 1.00.

From the first group, one patient (6.3%) was excluded from the study because of discontinuing the drug. In second group, 3 patients (15.8%), because of refuting reference, and one case (5.3%), because of rising of serum zinc level, were excluded from the study. So, in both of the two groups, 15 patients continued the study to the end of forth month. In the first group, out of 15 patients, one (6.3%) showed no response during 4 months of the study, considering with Fisher's exact test and P-value of 1.00, had no statistically significance.

Both of the two groups showed no response during the first month of the therapy. The mean of responses in the third and forth months, in the first group were 19.3% (± 9.3%) and 21.43% (± 11.6%), respectively and for the second group, were 20.8% (± 8.7%) and 24.7% (± 11.0%), respectively (Table 2). Although, the response in the second group were more than the first group, T-test revealed no statistically significant differences between the two groups, in the third and forth months with P-values equal to 0.6 and 0.4, respectively. To conclude, topical corticosteroid plus oral zinc sulfate had no preference on topical corticosteroid only.

**Table 2:** The mean of responses in third and forth months in the two drug-prescribed groups, who continued treatment till the end of the study

Group	Month	Number	Mean of response (%)	SD
First *	Third	15	19.13	9.36
Second**	Third	15	20.83	8.72
First	Forth	15	21.43	11.64
Second	Forth	15	24.70	11.04

* topical corticosteroid

** oral zinc sulfate-topical corticosteroid combination

In the view of the complication of zinc sulfate, only 2 (13.3%) patients of the second group complained of a little tolerable gastric burning.

## Discussion

Vitiligo is an acquired depigmenting disorder due to loss of melanocytes and the resultant absence of pigment production affecting skin and mucosal surfaces [5], with a prevalence of about 1-4% [22-24].

Although neither life threatening, nor symptomatic (except that depigmented patches burn easily when exposed to the sun) the effect of vitiligo can be cosmetically and psychologically devastating, resulting in low self-esteem, poor body image, and difficulties in sexual relationships [10,25]. It is a frustrating condition to treat, spontaneous repigmentation occurs in more than 15% to 25% of cases [12]. Sun protection of the vitiliginous areas with sunblocks is important [9,11], which help prevent sunburn and thus may lessen photodamage as well as the chance that a Koebner phenomenon will occur. Sunscreens also decrease tanning of the uninvolved skin and therefore lessen the contrast with vitiliginous lesions [4]. Cosmetic improvement can be achieved by camouflage products and self-tanning dyes [9].

Because the disease is still not understood, there is a plethora of different treatments including topical corticosteroids, calcineurin inhibitors, vitamin-D derivatives, phototherapy (ultraviolet [UV] A, narrowband UVB), photochemotherapy (psoralen plus UVA [PUVA], psoralen with sunlight [PUVAsol]), surgical techniques [4,7,10,14,18,26,27], excimer laser [4,7,9,14,18,26-28], topical prostaglandin E (PGE2) [7], and combinations of topical therapies and light treatment [10]. Complementary therapies have also been used, the most interesting being ginkgo biloba [10], and levamisole [29] which have been reported to have immune-modulating properties [10]. Pseudocatalase cream with Dead Sea climatotherapy are also compatible with repigmentation [10]. Topical fluorouracil [30], topical melagenina I and II, minoxidil [7], oral L-phenylalanine [10,31-34], homeopathy, ayurvedic medicine, climtologic, and balneologic therapies [7] are as alternative therapy for vitiligo.

Zinc is one of the important trace elements related to health and disease [35]. Essentiality of zinc is related mainly to its function as the metal moiety of important enzymes [3]. The most important of these processes are cellular respiration, cellular utilization of oxygen, DNA and RNA reproduction, maintenance of cell membrane integrity, and sequestration of free radicals [36].

Zinc in combination with other micronutrients such as copper, cobalt, nickel, iron, manganese, and calcium [21] plays an important role in the process of melanogenesis [3,21]. They catalyze the rearrangement of dopachrome to form 5,6-dihydroxy indole-2 carboxylic acid (DICA) [3,21], and enhancement of eumelanin polymer formation from monomers [21]. This process is at the final stage of eumelanin formation in melanogenesis [21].

The most frequent adverse effects of zinc salts given orally are gastrointestinal and include abdominal pain, dyspepsia, nausea, vomiting, diarrhea, gastric irritation, and gastritis [37].

There are few controlled trials assessing efficacy of natural health products (e.g. vitamins, minerals, herbal medicines and other supplements) for vitiligo, but those that have been published generally show weakly positive outcomes with few adverse reactions [14]. On the other hand, with searching the computerized bibliographic database Pub Med, we found no study of zinc efficacy in treatment of vitiligo. It appeared that our study is the first one to investigate zinc efficacy in the treatment of vitiligo.

Analysis of the zinc level in the study of Shameer *et al *revealed a reduced level in 21.6% of the patients. Only one patient showed elevated level of zinc. In this study, the serum zinc level in the control group was within the normal range. This differences between two groups was statistically significant (P < 0.0002) [3]. In another study, Arora *et al *showed that serum zinc was lower in vitiligo patients than control group, but this difference was not statistically important [20]. In our study, the serum zinc level were normal in 73 (84.9%), increased in 4 (4.7%), and decreased in 9 (10.5%) of the patients. Unfortunately, we had no control group for comparing the serum zinc level. In spite of these, our study compared with Shameer's one, revealed lower frequency of reduced serum zinc level and higher frequency of increased serum zinc level.

This study showed that the response to the oral zinc sulfate-topical corticosteroid combination was more than the topical corticosteroid alone, but T-test revealed no statistically significant difference between them.

## Conclusion

We conclude that topical corticosteroid plus oral zinc sulfate had no preference on topical corticosteroid only. Considering the more effect of corticosteroid plus zinc sulfate compared with corticosteroid alone, it appears that more robust long-term randomized controlled trials with more patients, maybe with higher doses of zinc sulfate, are needed to fully establish the efficacy of oral zinc in management of vitiligo.

## Competing interests

The authors declare that they have no competing interests.

## Authors' contributions

All the authors have read the article carefully and have approved this.

1 - RY. Designing the study, Supervising the trial process, gathering the related papers, gathering the patients, examining the patients, completing the questionnaire, writing the article, revising the completed article.

2 - MO. Designing the study, supervising the trial process, gathering the patients, examining the patients, completing the questionnaire, writing the article.

3 - NB. Designing the questionnaire, gathering the patients, examining the patients, completing the questionnaire, writing the article.

## Pre-publication history

The pre-publication history for this paper can be accessed here:

http://www.biomedcentral.com/1471-5945/11/7/prepub
